# Resolving the effect of wrist position on myoelectric pattern recognition control

**DOI:** 10.1186/s12984-017-0246-x

**Published:** 2017-05-04

**Authors:** Adenike A. Adewuyi, Levi J. Hargrove, Todd A. Kuiken

**Affiliations:** 1 Department of Biomedical Engineering, Northwestern University, Chicago, IL USA; 2 Center for Bionic Medicine, Shirley Ryan Ability Lab, 355 East Erie, Suite 11-1414, Chicago, IL 60611 USA; 3 Feinberg School of Medicine, Northwestern University, Chicago, IL USA; 4 Department of Physical Medicine and Rehabilitation, Northwestern University, Chicago, IL USA

## Abstract

**Background:**

The use of pattern recognition-based methods to control myoelectric upper-limb prostheses has been well studied in individuals with high-level amputations but few studies have demonstrated that it is suitable for partial-hand amputees, who often possess a functional wrist. This study’s objective was to evaluate strategies that allow partial-hand amputees to control a prosthetic hand while allowing retain wrist function.

**Methods:**

EMG data was recorded from the extrinsic and intrinsic hand muscles of six non-amputees and two partial-hand amputees while they performed 4 hand motions in 13 different wrist positions. The performance of 4 classification schemes using EMG data alone and EMG data combined with wrist positional information was evaluated. Using recorded wrist positional data, the relationship between EMG features and wrist position was modeled and used to develop a wrist position-independent classification scheme.

**Results:**

A multi-layer perceptron artificial neural network classifier was better able to discriminate four hand motion classes in 13 wrist positions than a linear discriminant analysis classifier (*p* = 0.006), quadratic discriminant analysis classifier (*p* < 0.0001) and a linear perceptron artificial neural network classifier (*p* = 0.04). The addition of wrist position data to EMG data significantly improved performance (*p* < 0.001). Training the classifier with the combination of extrinsic and intrinsic muscle EMG data performed significantly better than using intrinsic (*p* < 0.0001) or extrinsic muscle EMG data alone (*p* < 0.0001), and training with intrinsic muscle EMG data performed significantly better than extrinsic muscle EMG data alone (*p* < 0.001). The same trends were observed for amputees, except training with intrinsic muscle EMG data, on average, performed worse than the extrinsic muscle EMG data. We propose a wrist position–independent controller that simulates data from multiple wrist positions and is able to significantly improve performance by 48–74% (*p* < 0.05) for non-amputees and by 45–66% for partial-hand amputees, compared to a classifier trained only with data from a neutral wrist position and tested with data from multiple positions.

**Conclusions:**

Sensor fusion (using EMG and wrist position information), non-linear artificial neural networks, combining EMG data across multiple muscle sources, and simulating data from different wrist positions are effective strategies for mitigating the wrist position effect and improving classification performance.

## Background

The application of advanced signal processing and innovative surgical procedures has expanded the use of pattern recognition of electromyographic (EMG) signals to control prosthetic devices [1–3]. The majority of this work has focused on restoring function to individuals with high-level amputations, who make up less than 10% of all upper-limb amputees in the United States [4, 5]. Few studies have sought to apply pattern recognition control to individuals with partial-hand amputations, who constitute the majority of upper-limb amputees. Although often termed a “minor” amputation [5], the impact of partial-hand amputation on employment and self-image is increasingly recognized as being comparable to that of more proximal level amputations [6, 7]. Partial-hand amputations are difficult to treat effectively with a prosthesis [8–10] and cause individuals to perceive themselves as having a greater disability than those with higher level unilateral amputations [11, 12]. The recent introduction of externally powered, independently functioning digits, such as the i-limb quantum (Touch Bionics Inc.) and Vincentpartial (Vincent Systems GmbH), offer exciting possibilities for improving hand function of partial-hand amputees.

Partial-hand amputees often retain the ability to move their wrists, and preservation of residual wrist motion is critical for functional performance of everyday activities. With conventional myoelectric control, where an estimate of EMG amplitude is used for proportional control of an actuated joint, the prosthetist must use the EMG from the extrinsic hand muscles when intrinsic hand muscles do not provide viable control signals [8]. Since the forearm contains muscles that move both the fingers and the wrist, the user must generate EMG activity to control the prosthetic fingers without significant wrist movement, which may generate myoelectric signals that disrupt control [8]. One recent study showed that when non-amputees are limited to two degrees of freedom at the wrist (pronation/supination and flexion/extension) and 1° of freedom at the hand (open/close), they perform similarly to when they are limited to a 1°-of-freedom rotating wrist coupled with their natural 22°-of-freedom hand [13]. Thus, a clinically successful partial-hand pattern recognition control system must both provide high performance accuracy and allow the individual to retain use of their wrist.

Muscle contractions responsible for different wrist movements influence properties of the surface EMG recorded from the forearm during hand movements. Joint angle may also influence EMG patterns as a result of various internal physiological factors: changing the angle of the joint about which a muscle is fixed can alter muscle geometry and affect the relative positions of muscle fibers and motor units, not only with respect to themselves but also with respect to the skin surface electrodes [14]. Pattern recognition control depends on the user’s ability to generate repeatable and differentiable muscle contractions. Thus, changes in EMG patterns due to wrist position can degrade performance of the control system. Studies have shown that variations in arm position substantially impact the ability of pattern recognition control systems to classify hand grasps [15–17]. Our previous studies demonstrate that varying wrist position adversely affects pattern recognition performance in both offline and real-time virtual studies [18, 19]. We showed that the severity of this wrist position effect is diminished by training the classifier with data from multiple wrist positions and combining EMG data from the extrinsic and intrinsic muscles of the hand [18, 19], but these interventions do not reduce classification error to the level seen when the wrist is in one position.

To attenuate the limb position effect in individuals with higher level amputations, other studies have suggested that (i) adding information from a limb position sensor as an additional input into a pattern recognition system [16] and (ii) using a two-stage cascade classifier that uses a position sensor in the first stage for limb position identification and EMG for limb motion classification in the second stage may reduce the effect of limb position variation on classification performance [15, 16]. However, these approaches require the user to train the pattern recognition system by performing each hand motion in multiple limb positions. Since this laborious training process must be repeated whenever retraining is needed, it would be beneficial to be able to predict changes in EMG features as a function of wrist position, such that future retraining procedures would only require data collected in one wrist position. An ideal controller would thus be able to provide wrist position–independent control after being trained in one wrist position.

This work evaluates several strategies in non-amputees and partial-hand amputees for improving classification of hand grasps performed with varying wrist positions. In this study, we (1) evaluate the benefit of incorporating wrist position sensor information into linear and non-linear controllers and (2) propose a potential method for developing a control system that provides wrist position-independent control after being trained in one wrist position.

## Methods

### Data collection

Six non-amputees with no known neurological or physical deficits performed the experiments described in this study. Two partial-hand amputees-one with an amputation of all 5 fingers at the metacarpophalangeal joints (Subject 1) and one with a thumb amputation (Subject 2) -also performed the experiments. All subjects gave written consent for the collection of data, images and video recordings, and experiments were performed at the Rehabilitation Institute of Chicago under a protocol approved by the Northwestern University Institutional Review Board.

**Fig. 1:**
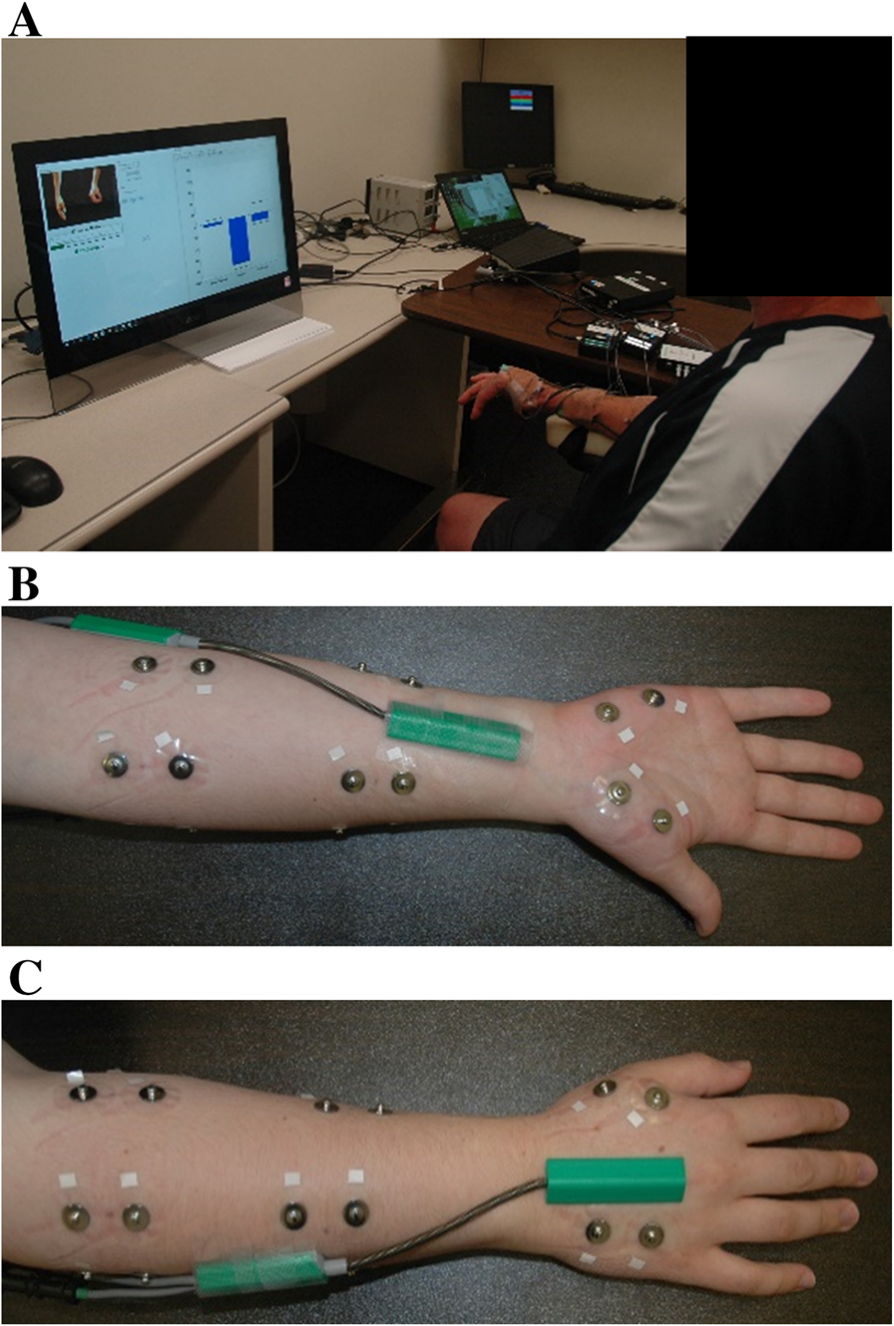
Experimental setup. **a** Subjects were prompted by a computer to perform each hand grasp in 13 wrist positions and received wrist position visual feedback. **b** and **c** anterior and posterior view, respectively, of a non-amputee subject depicting electrode and goniometer locations on forearm and hand

Nine self-adhesive bipolar surface Ag/AgCl EMG electrodes (Bio-Medical Instruments) were evenly spaced around the dominant forearm for non-amputees or residual forearm for amputees with an inter-electrode distance of 2.5 cm, with 5 electrodes on the proximal forearm, 2–3 cm distal to the elbow, and 4 electrodes on the distal forearm, 7–8 cm proximal to the wrist (Fig. 1). Four electrodes were placed on the hand: 2 electrodes on the palmar side and 2 electrodes on the dorsal side (Fig. 1). The ground electrode was placed on the olecranon of the elbow.

A biaxial flexible electrogoniometer (SG110, Biometrics Ltd) was used to record wrist flexion, extension, abduction and adduction. A single axis torsiometer (Q150, Biometrics Ltd) was used to record wrist pronation and supination. The distal end of the torsiometer was attached to the midline of the anterior forearm immediately proximal to the wrist joint and the proximal end of the torsiometer was attached to the forearm, immediately distal to the medial epicondyle of the humerus, in a position that did not interfere with the electrode placements on the forearm (Fig. 1b). The distal end of the biaxial goniometer was attached to the back of the hand, over the third metacarpal, such that it was parallel with the center axis of the hand and its proximal end was attached over the posterior midline of the forearm (Fig. 1c).

### Procedure

Subjects were prompted to position their wrist in one of 13 wrist positions. These positions were located at the end-range and mid-range of motion for flexion, extension, supination, pronation, abduction and adduction, in addition to a “neutral” wrist position. For the neutral position, subjects held their wrist at 0° in all 3° of freedom. Subjects received visual feedback of their wrist position from a computer monitor. For each wrist position, subjects were required to maintain the other two wrist degrees of freedom at 0° ± 5°. Subjects were visually prompted to perform one of 4 hand motions (chuck grasp, key grasp, an open hand posture, and a rest posture). Chuck and key grasps were chosen because they are the most common grasps used in activities of daily living [20]. Each hand posture was held for 3 s and repeated 6 times in each wrist position for a total of 78 repetitions per hand grasp.

To ensure that non-amputee subjects maintained the same pinch force throughout each grasp, subjects received visual feedback of pinch forces produced during chuck and key grips using an electronic pinch gauge (12–0023, Fabrication Enterprises). Subjects were required to maintain a grasp force that was 15–20% of their maximal voluntary grasp force made in a neutral wrist position; this force level was comfortable for all grasps in all wrist positions. To avoid fatigue, subjects were allowed 2–5 min rests between trials, where a trial consisted of 3 repetitions of the four hand postures in one wrist position.

### Signal processing

EMG signals were acquired using a custom built EMG amplifier with a total gain of 2000× (2× Hardware gain, 1000× Software gain) for each channel. All EMG data were digitally sampled at 1000 Hz using a custom-built A/D converter based on a TI AD1298 bioamplifier chip and band-pass filtered (30-350Hz) with a Type 1, 8th order Chebyshev digital filter. Goniometer data were sampled at 1000Hz with a custom-built 16 bit A/D converter and low-pass filtered at 10Hz with a 3rd order Butterworth filter.

### Data analysis

Offline analyses were performed using MATLAB 2015a software (The Mathworks, Natick, MA, USA). For all conditions, data were segmented into 200 ms windows with a 20 ms frame increment [21].

#### Effect of classifier type and wrist position on classification error

A combination of four EMG time-domain features (mean absolute value, number of zero-crossings, waveform length, and number of slope sign changes) and six coefficients of a 6th order autoregressive model (hereafter called TDAR features) were extracted from each EMG data window. For each window, the average value of the goniometer and torsiometer data was also calculated (hereafter called POS, for position features). Four classifiers, two linear and two non-linear, were compared: (1) a linear discriminant analysis classifier (LDA), (2) a quadratic discriminant analysis classifier (QDA), (3) a multilayer perceptron neural network with linear activation functions in its one hidden layer (LNN), and (4) a multilayer perceptron artificial neural network with nonlinear hyperbolic tangent sigmoid activation functions in its one hidden layer (MLPANN). The LDA was selected because it is the most commonly used for the classification of limb movements using EMG. It was compared to a QDA because they make very similar assumptions about the data except that it allows non-linear boundaries between data. These were compared to an LNN and MLPANN classifier as they make no assumptions about the data.

All classifiers were trained using data from (1) only extrinsic muscle EMG data, (2) only intrinsic muscle EMG data, or (3) a combination of all extrinsic and intrinsic muscle EMG data. Data were divided into training data sets (50% of all data), testing data sets (30% of all data) and validation data sets (20% of all data). Each classifier was evaluated using two-fold cross-validation with these sets. The validation data set was used to minimize overfitting of the neural networks; training of the neural networks stopped once the error of the validation sets began to increase. Seven hidden layer neurons were empirically chosen for the MLPANN, and the LNN had four neurons in its hidden layer. Since the LNN has linear activation functions, it simply maps the weighted inputs to the output of each neuron and is thus mathematically equivalent to a reduced two-layer input-output model [22]. The neural networks were trained using scaled conjugate gradient descent [23]. This analysis was performed with two feature sets, (1) the TDAR feature set alone and (2) the TDAR combined with the POS feature set.

An exhaustive search was performed to determine the optimal number of wrist positions needed for classifier training. An LDA classifier was trained using data from 1 to 13 wrist positions and tested on data from all 13 wrist positions. All possible combinations of data from *n* wrist positions were evaluated, and the combination with the lowest error was chosen for each subject and plotted as a function of number of wrist positions. For example, when the number of wrist positions chosen was 4, the four best positions that yielded the highest classification accuracy for each subject was evaluated.

To determine if position-specific classifiers can perform better than one generalized classifier trained with data from all wrist positions, two training paradigms were evaluated. In training paradigm 1, one classifier was trained with data from all wrist positions and tested with data from each wrist position separately, with the results averaged across positions. In training paradigm 2, thirteen classifiers were trained and tested with data from each wrist position separately and results were averaged across classifiers.

#### Predicting changes in feature as a function of wrist position

**Fig. 2:**
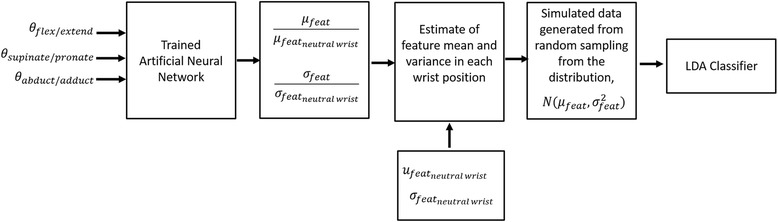
A neural network was trained to predict changes in the mean and variance of each EMG feature, from each channel, for each class as a function of the mean and variance of the feature in a neutral position. The output of each neural network was used to generate data to simulate real data collected from all wrist positions. This simulated data was then used to train an LDA and tested using real data

To predict how each feature changes as a function of wrist position, a neural network was used for non-linear regression. The neural network had 3 inputs which were the wrist position in each of the three degrees of freedom. The network had 3 neurons in its one hidden layer with hyperbolic tangent sigmoid activation functions and 1 output neuron with a linear activation function. The neural network was trained using scaled conjugate gradient descent. A separate neural network was trained for each feature, from each channel, for each class. Fifty percent of the data from each wrist position was used to calculate the mean and variance of each feature in each position, which were then divided by the mean or variance, respectively, of each feature in a neutral wrist position. The neural network was then trained to predict the change in mean or variance of each feature (Fig. 2), where 20% of the data was used for cross-validation and 30% was used for testing. The coefficient of determination, *r* ^*2*^, was calculated to measure the performance of each neural network.

Three data sets were compared: (1) the real dataset, (2) a simulated dataset generated by randomly sampling from distributions described by the means and variances generated by the neural network and (3) a simulated dataset generated by randomly sampling from distributions described by the mean and variance generated by the neural network for *only* the neutral wrist position. The three datasets were used to train three LDA classifiers, which were tested using the real dataset. The number of data points used in all simulated datasets was equivalent to the number of data samples in the original real data set. For this analysis, only TDAR features were evaluated, and the LDA classifier was used to determine average classification error across subjects.

To summarize, the inputs into the neural network were the wrist position angles and the outputs were either the mean or variance of each feature for each wrist position *relative* to the same feature’s mean or variance in a neutral wrist position. Thus, once trained, the neural network is able to predict the mean and variance of each feature in each position with *real* data collected from *only one* neural wrist position. In other words, by using this method, one would only need to perform the grasps in all other wrist positions *once*, use this data to train the neural network and simulate the data that will be generated in other wrist positions. Once this is complete, is no longer a need to continually monitor wrist position.

### Statistical analysis

To determine the effect of classifier type and wrist position on classification error, a three-way repeated measures analysis of variance test (ANOVA) was performed with subject as a random effect, and muscle set, feature set, and classifier type as fixed effects. A two-way repeated measures ANOVA, with subject as a random effect and muscle set and training paradigm as fixed effects, was used to determine the effect of training paradigm on classification performance. To test the performance of the simulated datasets, a two-way ANOVA test was performed with subject as a random effect and muscle set and data set as fixed effects. All post-hoc comparisons were made using a Bonferroni correction factor to determine significance. All statistical analyses were performed using Minitab 16.2.4 (Minitab Inc. PA, USA), and the significance level was set at 0.05. Statistical analyses were performed only on non-amputee data.

## Results

### Effect of classifier type, muscle set and wrist position information on classification

**Fig. 3:**
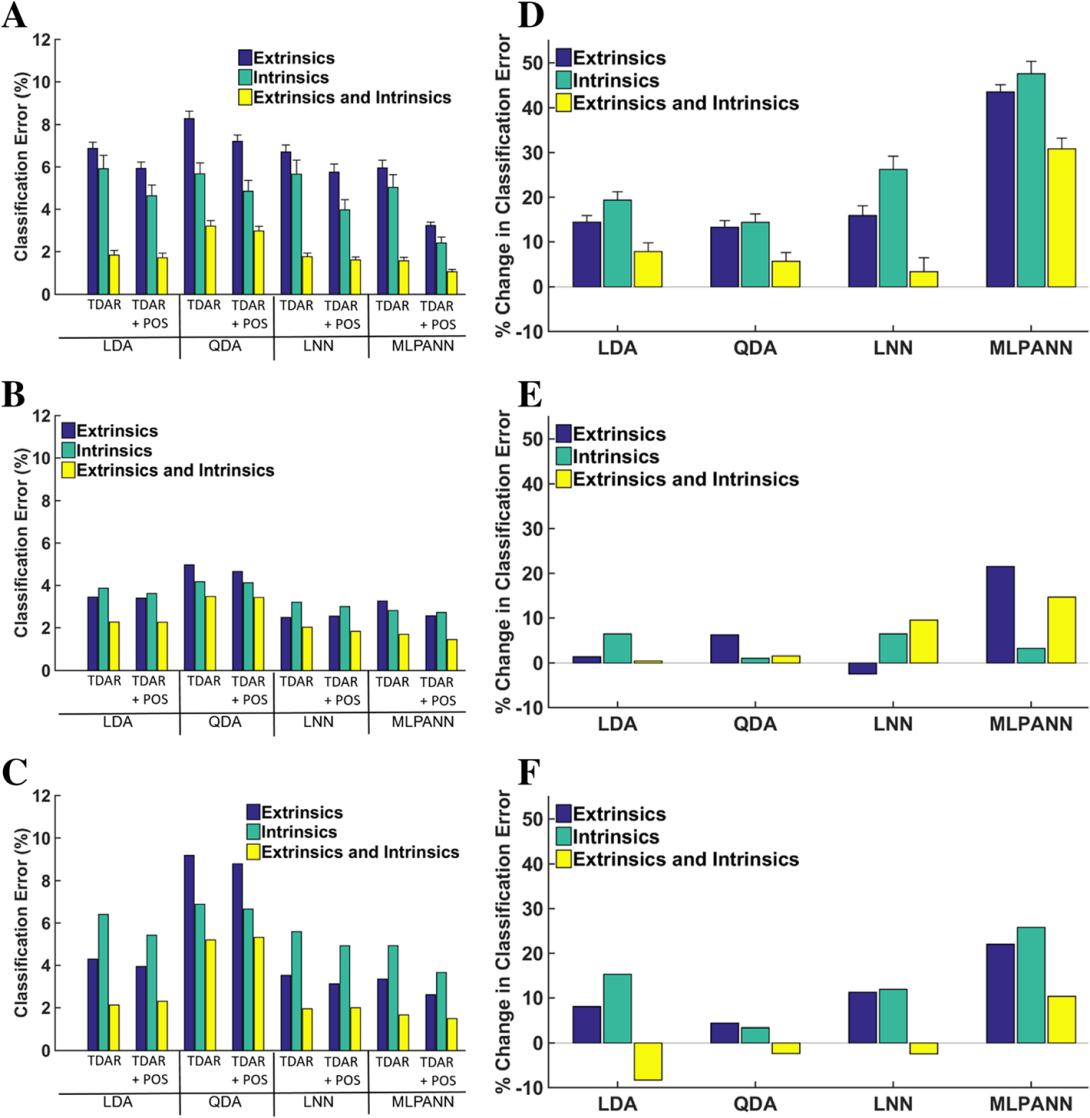
Effect of wrist position information on linear and non-linear classification of 4 hand motions in 6 non-amputees and 2 partial-hand amputees. Each classifier was trained and tested using data from 13 wrist positions. Each classifier was trained with either EMG features alone (TDAR) or EMG features combined with wrist position features (TDAR + POS). Results are shown for (**a**) non-amputees, (**b**) Partial-hand subject 1 and (**c**) Partial-hand subject 2. The percent change in classification error when wrist position data was combined with EMG features is shown in figures. D-F. A positive change represents an improvement in performance with the addition of wrist position features. Results are shown for (**d**) non-amputees, (**e**) Partial-hand subject 1 and (**f**) Partial-hand subject 2. Error bars represent standard errors. LDA: Linear discriminant analysis; QDA: Quadratic discriminant analysis; LNN: Neural Network with linear activation function; MLPANN: Neural Network with non-linear activation functions

Figure 3 shows the effect of three factors (muscle set, feature set, and classifier type) on classification error. For non-amputees (Fig. 3a), there was a significant main effect of all three factors (*p* < 0.001). No interaction terms were found to be significant. The use of wrist position information as an additional feature improved relative performance for the LDA, QDA, and LNN classifiers by 14, 13 and 16% for the extrinsic muscle data, 19, 14, and 26% for the intrinsic muscle data and 8, 6, and 3% for the combination of extrinsic and intrinsic muscle data, respectively. The addition of wrist position information had a much greater effect on the performance of the MLPANN, improving relative error by 43% for the extrinsic muscle, 48% for the intrinsic muscles and 30% for the combination of extrinsic and intrinsic muscle data (Fig. 3d) with absolute improvements in error of up to 5.4%. Pairwise comparisons showed that the MLPANN performed significantly better than the LDA (*p* = 0.006), QDA (*p* < 0.0001) or LNN (*p* = 0.04) classifiers, and the LNN performed significantly better than the QDA (*p* = 0.02). The combination of extrinsic and intrinsic muscle data performed significantly better than using intrinsic (*p* < 0.0001) or extrinsic muscle data alone (*p* < 0.0001), and intrinsic muscle data performed significantly better than extrinsic muscle data (*p* < 0.001). For partial-hand subjects 1 and 2, similar trends were observed: the LDA, QDA and LNN resulted in small changes in classification error with the inclusion of wrist position information, and the MLPANN classifier performed the best. The QDA was the worst performing classifier for both amputees, and the combination of extrinsic and intrinsic muscle data performed better than the two muscle group data sets alone. In contrast to non-amputees, for both amputee subjects the intrinsic muscle data, on average, performed worse than the extrinsic muscle data.

**Fig. 4:**
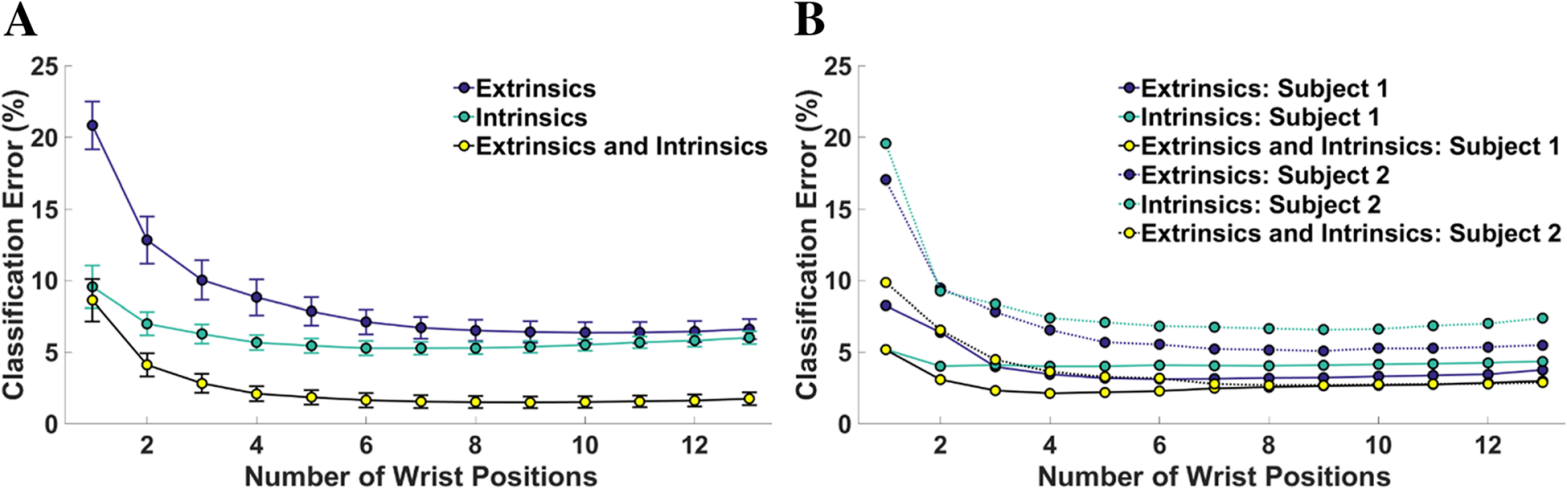
Classification error for 4 hand motion classes as a function of number of wrist positions used to train the classifier for (**a**) 6 non-amputees and (**b**) 2 partial-hand amputees. Error bars represent standard error

**Fig. 5:**
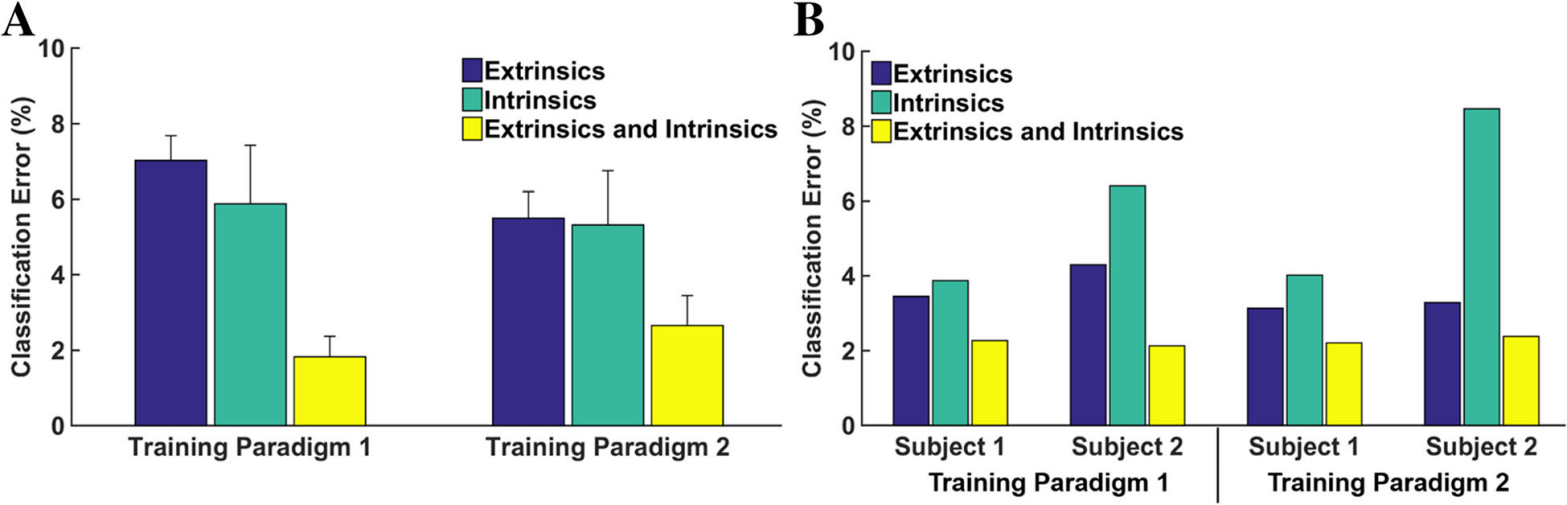
Classification error for 4 hand motion classes for (**a**) 6 non-amputees and (**b**) two partial-hand amputees. In training paradigm 1, one classifier was trained with data from all wrist positions and tested with data from each wrist position separately. In training paradigm 2, thirteen classifiers were trained and tested with data from each wrist position separately and the results of all 13 classifiers were averaged. Error bars represent standard error

Figure 4 shows the relationship between the number of wrist positions included in the training data and classification error. Error decreases substantially at the beginning of the curve as more wrist positions are added, but there is no further significant decrease in error for the extrinsic when more than 9, 4, or 6 wrist positions are included, for the extrinsic (*p* = 0.39), intrinsic (*p* = 0.14), or combination of extrinsic and intrinsic muscle data (*p* = 0.13), respectively. Similar trends were observed for amputee subjects. We found no statistically significant difference in average classification error between a classifier trained with data from all wrist positions and tested with data from each wrist position separately and 13 classifiers trained and tested with data from each wrist position separately (*p* = 0.47) (Fig. 5).

### Predicting changes in feature as a function of wrist position

**Fig. 6:**
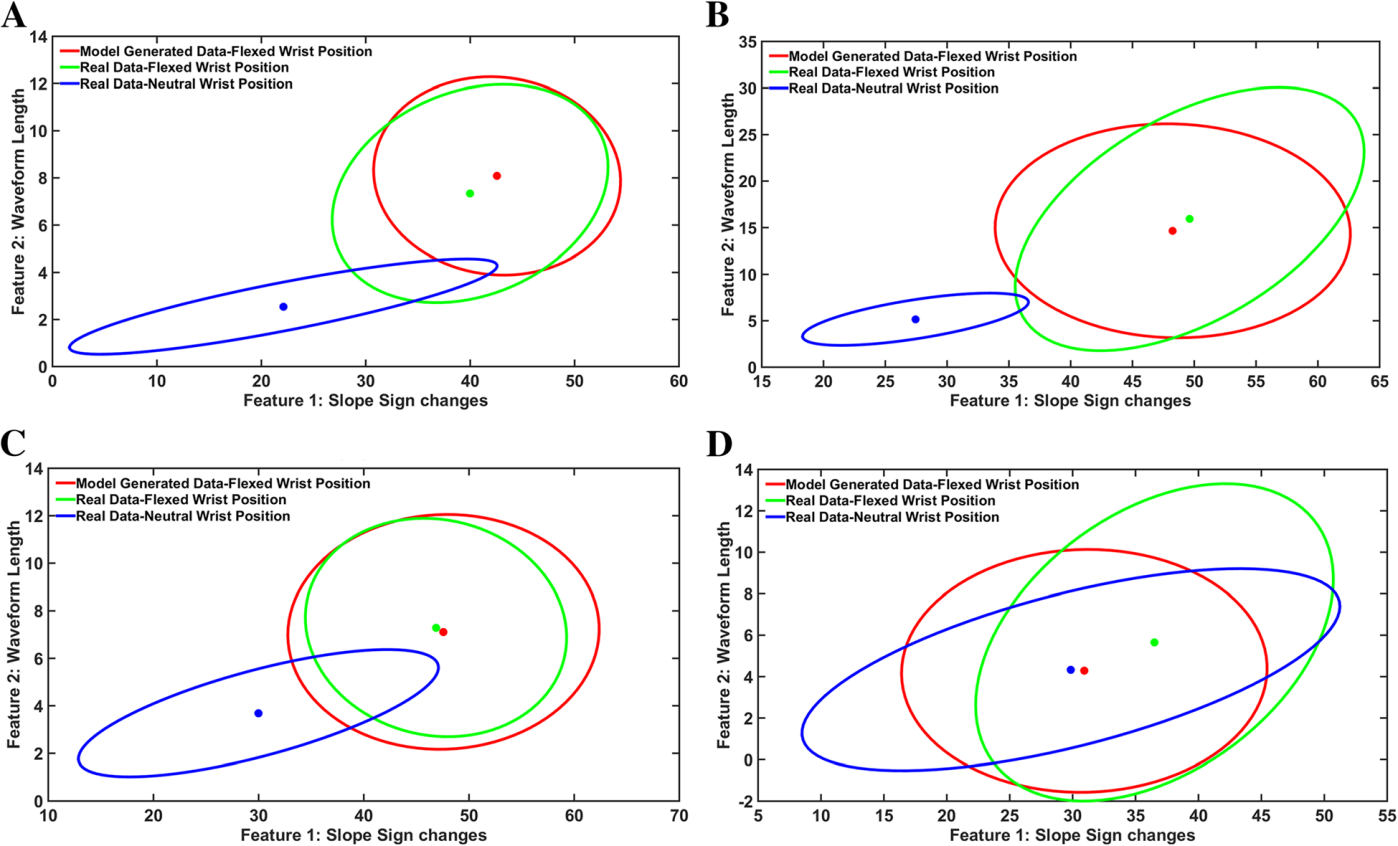
Representative plots from one subject of the estimation of the mean and variance of two features (slope sign changes and waveform length) from one channel when the wrist is in a neutral position and when the wrist is flexed. **a** No hand motion class (**b**) Hand open class (**c**) Chuck grip class (**d**) Key Grip class. Each dot within an ellipse represents the mean of the features. Each ellipse is constructed using the mean and the two standard deviation vectors

The neural networks were able to accurately predict the change in mean of each feature in each wrist position relative to the mean of the respective feature in a neutral wrist position. Figure 6 shows a representative plot depicting the mean and variance for two features, waveform length and slope sign changes when the wrist is in a neutral position and when the wrist is in a flexed position (Fig. 6). On average, for non-amputees, the *r* ^*2*^ values were 0.84 for extrinsic muscle data, 0.82 for intrinsic muscle data and 0.83 for the combination of extrinsic and intrinsic muscle data. For the amputee subjects, the *r* ^*2*^ values were on average 0.79, 0.73 and 0.77 for the extrinsic, intrinsic, and the combination of extrinsic and intrinsic muscle data, respectively (Table 1). The neural network was less able to predict the variance of the features. The *r* ^*2*^ values for non-amputees and amputees, respectively, were 0.55 and 0.6 for the extrinsic muscle data, 0.54 and 0.57 for the intrinsic muscle data and 0.55 and 0.59 for the combination of extrinsic and intrinsic muscle data (Table 2).

**Table 1:** Summary of the *r* ^*2*^ values for estimating the mean of each feature as a function of wrist position

		Non-Amputees	Amputees	
Muscle Set	Grasp	Mean ± SD	Subject 1	Subject 2
Extrinsics	No Movement	0.898 ± 0.04	0.836	0.836
	Hand Open	0.812 ± 0.04	0.719	0.806
	Key Grip	0.831 ± 0.03	0.755	0.862
	Chuck Grip	0.825 ± 0.05	0.777	0.73
Intrinsics	No Movement	0.913 ± 0.05	0.845	0.847
	Hand Open	0.837 ± 0.07	0.677	0.712
	Key Grip	0.771 ± 0.06	0.743	0.796
	Chuck Grip	0.74 ± 0.07	0.612	0.621
Extrinsics and Intrinsics	No Movement	0.902 ± 0.04	0.839	0.839
	Hand Open	0.819 ± 0.05	0.706	0.777
	Key Grip	0.813 ± 0.03	0.752	0.842
	Chuck Grip	0.799 ± 0.05	0.727	0.697

**Table 2:** Summary of the *r* ^*2*^ values for estimating the variance of each feature as a function of wrist position

		Non-Amputees	Amputees	
Muscle Set	Grasp	Mean ± SD	Subject 1	Subject 2
Extrinsics	No Movement	0.576 ± 0.06	0.701	0.707
	Hand Open	0.486 ± 0.09	0.569	0.602
	Key Grip	0.572 ± 0.05	0.527	0.615
	Chuck Grip	0.56 ± 0.06	0.536	0.539
Intrinsics	No Movement	0.652 ± 0.05	0.73	0.646
	Hand Open	0.507 ± 0.09	0.527	0.57
	Key Grip	0.504 ± 0.1	0.423	0.69
	Chuck Grip	0.512 ± 0.1	0.456	0.523
Extrinsics and Intrinsics	No Movement	0.6 ± 0.05	0.71	0.688
	Hand Open	0.493 ± 0.06	0.557	0.592
	Key Grip	0.551 ± 0.06	0.495	0.638
	Chuck Grip	0.545 ± 0.07	0.511	0.534

**Fig. 7:**
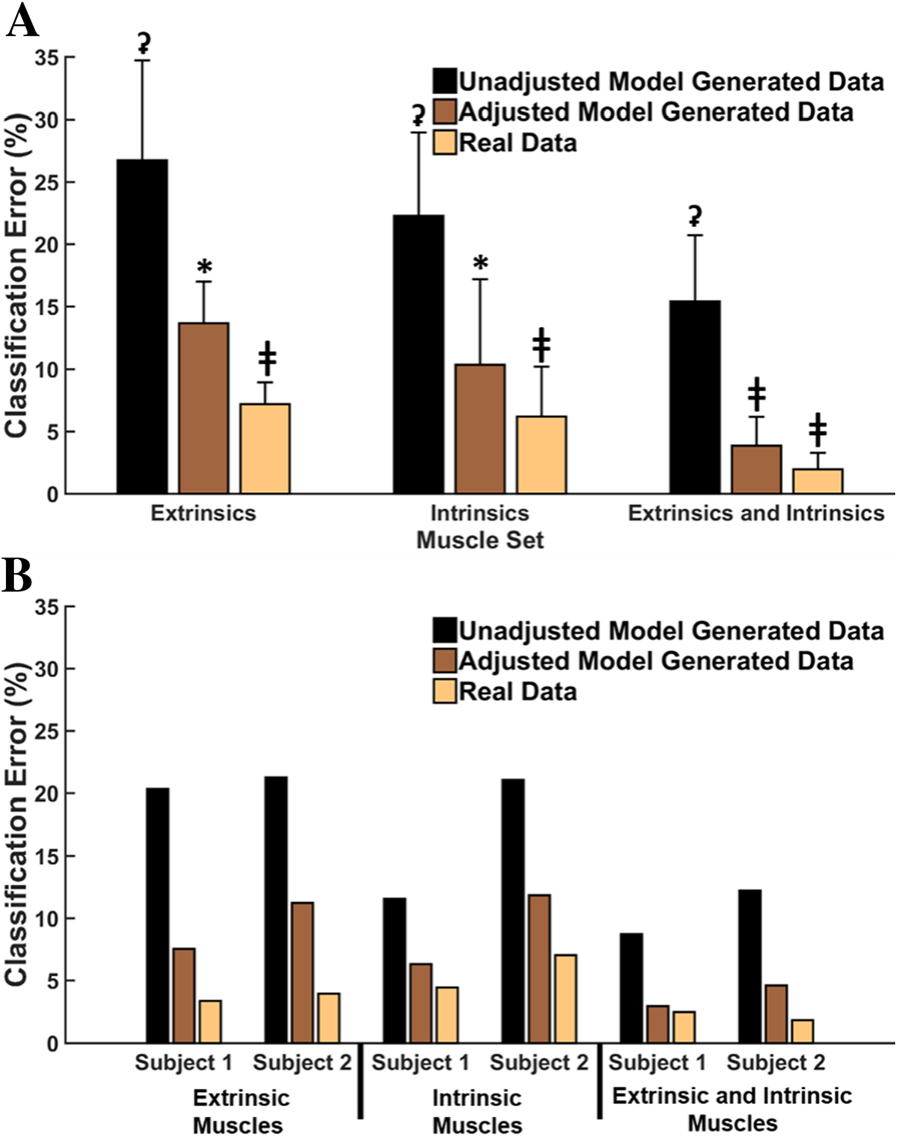
Three classifiers were trained using three datasets: (1) unadjusted model-generated data which was a simulated dataset generated by randomly sampling from distributions described by the mean and variance generated by the neural network for *only* the neutral position; (2) adjusted model-generated data which was a simulated dataset generated by randomly sampling from distributions described by the means and variances generated by the neural network from all positions; and (3) the real data set. **a** Results from non-amputee subjects; for each muscle set, datasets that are significantly different from each other do not share the same symbol. **b** Results from two partial-hand amputee subjects. Error bars represent standard deviation

Training the LDA classifier with real data from *only a neutral* wrist position and testing with real data from *all positions* resulted in high errors of 27, 22 and 15%, for extrinsic muscles, intrinsic muscles and the combination of both sets of muscles, respectively. However, when the LDA was trained with simulated data from all wrist positions, the error significantly decreased for all three muscle groups (Fig. 7a); by 48% for extrinsic muscle data (*p* < 0.001), by 54% for intrinsic muscle data (*p* < 0.001), and by 74% (*p* < 0.001) for combined data from both muscle groups. The same trends were observed in both partial-hand amputees, where error decreased by 63 and 47%, 45% (Subject 1) and 44%, 66 and 63% (Subject 2) for the extrinsic, intrinsic, and the combination of extrinsic and intrinsic muscle data, respectively (Fig. 7b). Moreover, there was no significant difference between the performance of the adjusted simulated data set and the real data set (*p* = 0.76) when data from the extrinsic and intrinsic muscles were combined.

## Discussion

This work sought to evaluate strategies that mitigate the effect of varying wrist position on pattern recognition classification of hand grasps, to facilitate the application of pattern recognition control to externally powered myoelectric partial-hand prostheses. We evaluated the benefit of incorporating wrist position sensor information into linear and non-linear controllers and established a control system that is capable of providing wrist position–independent control after being trained in one wrist position.

Overall, the performance of the LDA, LNN, and MLPANN classifiers were comparable for amputees and non-amputees, although the QDA performed worse than all other classifiers. This is likely because unlike the LDA, the QDA is a more complex model that allows for the heterogeneity of covariance matrices for each class of data. Consequently, it requires more data to estimate more parameters and its poorer performance may be attributed to overfitting of the training data. Though the use of wrist position information as an additional feature significantly improved performance across all classifiers, a multi-layer perception was better able to utilize the additional wrist position information, improving performance by 30–48%. The LDA is commonly used because it provides a good balance between classification performance and computational efficiency and performs as well as the MLPANN and LNN [2]. However, these studies do not consider classification performance for multiple wrist positions because they focus on applications to individuals with more proximal amputations. Our results suggest that for controlling partial-hand prostheses in multiple wrist positions, the benefit of wrist position information is best realized when it is incorporated into a multi-layer perception neural network.

In agreement with our previous studies [18], we found that combining extrinsic and intrinsic muscle data consistently resulted in significant improvement in performance over extrinsic or intrinsic muscle data alone for amputees and non-amputees. For non-amputees, training with intrinsic muscle EMG data alone performed significantly better than training with extrinsic muscle EMG data alone. However, for the amputee subjects, the extrinsic muscles generally performed better than the intrinsic muscles. It is worthwhile to note that it is clinically difficult to stably record from the intrinsic muscles. Moreover, 93% of partial-hand amputations are due to traumatic injury [5] and as such, the intrinsic hand muscles may be damaged or absent rendering them unsuitable as an EMG signal source. The differences between non-amputee and amputee performance may be due to damage to the intrinsic muscles of amputee subjects. Though there is high degree of variability in partial-hand amputations, the extrinsic muscles of partial-hand amputees are relatively intact and our results demonstrate that it is feasible to achieve control that is comparable to that of non-amputee subjects with extrinsic muscle EMG data.

In an attempt to resolve the limb position effect, Fougner et al. [16] proposed a two-stage position-aware classifier where the limb position was first detected and then a classifier specific for that position was used for motion classification. Our analysis showed when we compare a classifier trained with data collected from all wrist positions to the performance of the average of 13 wrist position specific classifiers, there are small, nonsignificant changes in offline classification error. In our analysis we did not classify wrist position. Thus even if a system had perfect 100% wrist position classification accuracy, the use of a position sensor would provide no benefit when used for two-stage classification.

Because training in multiple wrist positions can be burdensome for the user, it is important to minimize the number of wrist positions necessary to train the control system. Though classification performance generally improved with each additional wrist position, there was small improvement after more than 6 positions were included. In some instances, including data from too many wrist positions may increase error (e.g., an increase in the number of wrist positions from 6 to 13 increased classification error from 5.3 to 6, 4 to 4.4% and from 6.8 to 7.4% for the intrinsic muscle data for non-amputees, Subject 1, and Subject 2 respectively). This is likely because data from one wrist position had class labels that directly contradicted class labels from another wrist position. We further analyzed the data to determine if the classification of hand grasps from all wrist positions was better when the classifier was trained with the wrist positions in the mid-range of motion or at the end-ranges of motion and found that there was no significant difference between the two training sets. These findings suggest that the number of training positions is more important than the training position.

The previously discussed strategies require the collection of data from different wrist positions, which can be quite time consuming and possibly fatiguing for the user, especially when retraining of the control system is necessary. Ideally, a controller would be able to provide wrist position-independent control after being trained in one wrist position. By using a neutral network for non-linear regression, we demonstrate that it is feasible to accurately predict how EMG data features change as a function of wrist position, and thus we can use data collected from a neutral wrist position to generate simulated data for all wrist positions. Here, we used an artificial neural network to implement a “black box” approach, which does not consider the individual factors that could be contributing to the wrist position effect such as the changes in muscle length, moment arms, electrode position relative to the innervation zone, or muscle fiber recruitment [24]. Alternatively, one could use other approaches such as a biomechanical model that can model the effects of changes in musculoskeletal geometry on muscle activation patterns and muscle force. However, by using a black-box approach, we forego the complexities and challenges associated with such models. For example, the moment arms for the extrinsic hand muscles used in musculoskeletal models are based on results from cadaver studies which assume the same proximal muscle origins and insertions across subjects [25], but for partial-hand amputees, the insertions of the extrinsic muscle tendons would be highly variable, depending on each individual’s surgical procedure.

These results are limited in that the training and testing data sets are from the same day and experimental session. Though pattern recognition control deteriorates when classifiers are trained and tested with data collected from different days or sessions, a recent study has shown that between-day performance improves and approaches within-day performance when subjects perform contractions over 11 consecutive days [26]. These results imply that subjects are better able to make more consistent contractions when training over multiple days. It is thus possible that the mapping between EMG features and wrist position will be stable if subjects are trained over multiple days. Further multi-day experiments are needed to determine if the neural network maintains its performance across sessions.

One important consideration regarding the neural network regression model is that we assume each feature is independent and thus the change in feature as a function of wrist position is predicted separately for each feature. Consequently we lose any some mutual information across the features. Even with this loss of information, the performance using the model-generated data particularly with intrinsic and extrinsic muscles performs just as well as the real data set, implying that the issue is not critical. Perhaps this is because there are enough data from enough features to overcome this. It is possible however, that preserving the relation and covariability between features would better allow the model-generated data to more accurately predict the feature changes and improve performance.

Another potential limitation is that the analyses were performed offline and with only 4 hand motion classes (2 grasps, hand open and no movement). We expect classification error to increase when more hand grasps are available to the classifier though future work is needed to evaluate the extent to which wrist position information improves error and to determine if the performance of the simulated dataset generalize to more grasps. The relationship between offline error and real-time performance is unclear. Some previous research has demonstrated a minimal correlation between offline performance and usability with a virtual task [27, 28]; however other studies have shown significant correlation between offline classification error and real-time control [21, 29]. Though the findings of this study are promising, further real-time experiments in a virtual environment or with a physical prosthesis are warranted.

## Conclusion

The application of pattern recognition technology to control externally powered partial-hand prostheses offers exciting opportunities for restoring hand function. This study evaluated strategies that would promote this application while allowing a partial-hand amputee to retain residual wrist function. In this study, we compared the performance of linear and non-linear control strategies, and we also evaluated the benefit of adding information from a wrist position sensor to EMG data for improving pattern recognition control of hand grasps in multiple wrist positions. We found that adding wrist position information improved performance when incorporated into a neural network classifier for both amputees and non-amputees. We also successfully used non-linear regression to model the relationship between EMG features and wrist position and exploited this relationship to significantly improve performance of a control system trained with real data from one wrist position and tested with real data from multiple wrist positions.

## Abbreviations

ANOVA: Analysis of Variance
EMG: Electromyography
LDA: Linear Discriminant Analysis
LNN: Linear Neural Network (Neural Network with linear activation functions)
MLPANN: Multilayer Perception Artificial Neural Network (Neural Network with non-linear activation functions)
POS: (Wrist) Position Features
QDA: Quadratic Discriminant Analysis
TDAR: Time Domain and Autoregressive Features
